# Study on the Mechanism of Interfacial Friction Heating in Polymer Ultrasonic Plasticization Injection Molding Process

**DOI:** 10.3390/polym11091407

**Published:** 2019-08-27

**Authors:** Tao Peng, Bingyan Jiang, Yang Zou

**Affiliations:** 1 School of Mechanical and Electrical Engineering, Lushan South Road 932, Changsha 410083, China; 2 State Key Laboratory of High Performance Complex Manufacturing, Central South University, Lushan South Road 932, Changsha 410083, China

**Keywords:** ultrasonic plasticization injection molding, friction heating, ultrasonic vibration, finite element simulation

## Abstract

Ultrasonic Plasticization Injection Molding (UPIM) is an effective way to manufacture polymeric micro parts and has great potential for energy saving with processing polymeric materials of a small amount. To better control the UPIM process and improve the quality of micro parts, it is necessary to study the heat generation mechanism. In this paper, the interfacial friction heating process of UPIM was studied by finite element (FEM) simulation and experiment, and the temperature change in the friction interface was estimated. Then, the effects of different process parameters such as ultrasonic frequency and ultrasonic amplitude on the friction heating process were analyzed. The results showed that the rising trend of friction heating temperature was transient (finished within 1 s), and the change trend of FEM simulation was consistent with experimental results. Adjusting ultrasonic frequency and amplitude has a significant influence on the friction heating process. Increasing the ultrasonic frequency and amplitude can improve the efficiency of friction heating.

## 1. Introduction

Ultrasonic vibration energy has been widely applied in the polymer micro parts molding process, such as ultrasonic patterning [1], ultrasonic welding [2], ultrasonic bonding [3], ultrasonic imprinting [4,5], and ultrasonic plasticization injection molding (UPIM) [6,7]. Under the action of ultrasonic vibration, the intermolecular force of the polymer to be processed is weakened, the activity of the chain is enhanced, and the degree of chain entanglement is reduced, meanwhile, it is continuously softened [8]. Since the external friction energy among the polymer interface and the internal friction energy among the polymer chains are converted into heat, external heat sources will not be necessarily needed. Ultrasonic energy is used to plasticize polymer pellets in the UPIM process. UPIM has developed for its unique potential in materials and energy efficiency [9,10] and is gradually becoming an effective processing technology for the preparation of polymer micro-mold samples in recent years [11]. Compared to micro injection molding, UPIM does not exhibit significant thermal degradation when molding polymers and has good energy-saving effects [12].

For the UPIM process, studying the plasticization mechanism, controlling the plasticization process, and improving the quality of the injection molded parts are the focus of research. In the UPIM process, ultrasonic vibrations are transmitted to the polymer through the ultrasonic horn during the entire plasticization process. [13,14]. One of the challenges of the UPIM process is that only trace amounts of polymer are plasticized during the entire process. The whole ultrasonic energy is concentrated, and the material melting is very sensitive to the plasticization quality. Uneven plasticization and polymer degradation often occur when the UPIM process is improperly handled [9,11]. Plasticization process, processing parameters, and material properties directly affect the quality of micro parts, so scholars have carried out a lot of theoretical and experimental studies to clarify the mechanism of ultrasonic plasticization. In the UPIM process, there are two kinds of heat generation mechanisms: the friction heating process between the polymer particles contact interface and the viscoelastic heat generation process of the polymer [7,8,13,14]. Understanding the heat generation mechanism and predicting the temperature change during the UPIM process is of great significance for controlling the quality and improving the precision of plastic micro parts.

In our previous research, the friction heat generation process of the polymer particles interface was studied [15]. It was found that the friction heat generation process only occurred in the initial stage and existed for a short time during the whole plasticization process. Later, the viscoelastic heat generation process of the amorphous polymer during UPIM was studied by theoretical simulation and experiment [16]. The effects of parameters such as initial temperature, ultrasonic frequency, and ultrasonic amplitude on the viscoelastic heat generation were studied. The results showed that the initial temperature of the polymer had a significant effect on the rate of viscoelastic heating. The mechanism of heat generation during ultrasonic plasticization was studied in the aforementioned two studies.

Up to now, a few scholars have reported on the heat generation mechanism of ultrasonic plasticization of polymers. However, only the mechanism of polymer ultrasonic welding has been reported. The heat generated by ultrasonic welding is mainly confined to the interfacial area. Because of the differences in the arrangement of the ultrasonic horn, the direction of vibration propagation and the contact form of the polymer, the heating process of ultrasonic welding is different from that of the UPIM process. Relevant research on the ultrasonic processing of polymer is as follows: based on the viscoelastic theory, the mechanism of heat generation in ultrasonic welding of plastic torsional vibrator was studied in [17]. Friction heat generation and viscoelastic heat generation in ultrasonic welding of plastic were calculated, but the theoretical model lacked experimental verification. Literature [18,19,20,21] has involved the study of the viscoelastic heat generation mechanism in the process of ultrasonic welding through theoretical modeling and an experimental research. Based on the generalized Maxwell model, Arrhenius model, and semi-empirical WLF (Williams Landel Ferry) model, the viscoelastic heating phenomena were numerically simulated and solved by commercial FEM software ANSYS. The heat generation rate was calculated quantitatively. The mechanism of the influence of initial temperature, ultrasonic amplitude, and ultrasonic frequency on the heating rate was studied, but the friction heating process was not studied.

There are still many problems to be solved in the research of UPIM mechanisms, such as establishing the mathematical model of friction heat generation to estimate the temperature of the friction zone, and the mechanism of the influence of the friction and viscoelastic heat generation process on the performance and quality of micro parts. In this paper, we focused on the study of the friction heat process of cylindrical constant angle inclined polymer during the UPIM process. Based on FEM simulation and experiment, the friction heating mechanism of the UPIM process was analyzed, and the effects of different process parameters on the temperature of the friction zone were studied.

## 2. Materials and Methods

### 2.1. Physical Model

**Figure 1:**
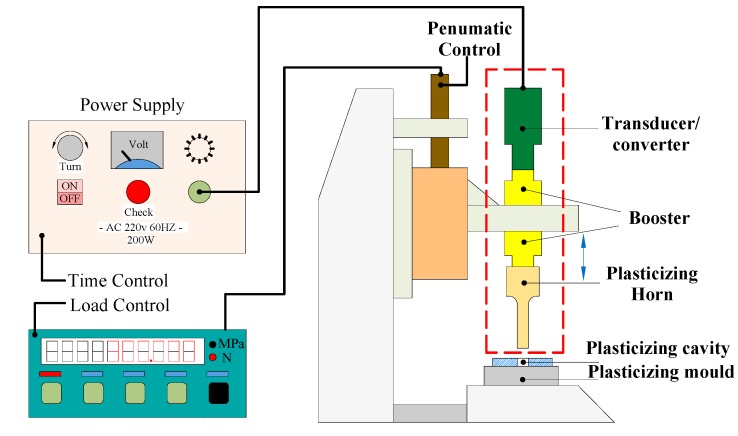
Equipment system of the ultrasonic plasticization injection molding process.

Ultrasonic plasticization injection molding (UPIM) can effectively increase the fluidity of polymer melt, reduce the extrusion pressure, and improve the properties of products by applying ultrasound technology to polymer micro-injection molding. The equipment system of the UPIM process is shown in Figure 1, which includes an ultrasonic power supply, pneumatic control unit, and load control unit. The UPIM process uses ultrasonic vibration energy to plasticize thermoplastic polymer particles to achieve a polymer filling cavity molding. The mechanical energy generated by ultrasonic vibration is converted to the heat of polymer particles, which includes two parts: friction heat generation and viscoelastic heat generation. Repeated vibration of the ultrasonic horn causes a shearing action between polymer particles. Friction work is done and part of the friction work is transformed into heat, then the friction interface of the polymer particles is softened locally. With continuous high-frequency vibration, the polymer particles melt because of viscoelastic heating and the rising temperature. The ultrasonic horn is also used as a plunger to push polymer melt into the cavity.

**Figure 2:**
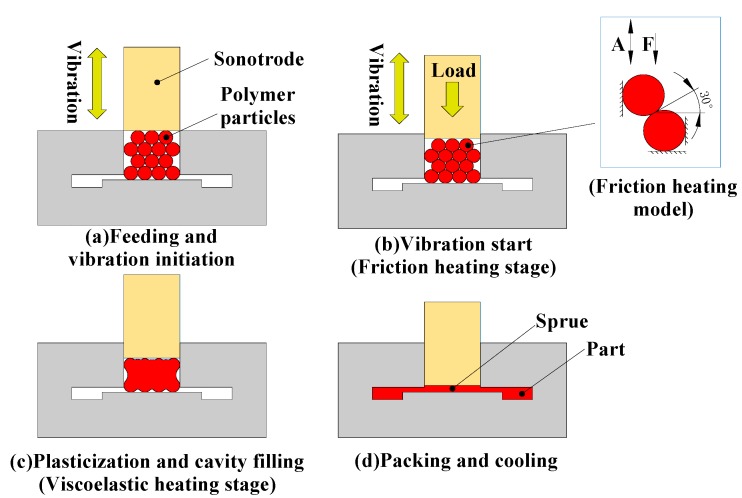
Principle of the ultrasonic plasticization injection molding Process.

The UPIM process can be divided into four stages, as shown in Figure 2. (a) Initialization of feeding and vibration: polymer particles are loaded into the plasticizing chamber, the position of the ultrasonic horn is controlled, and the polymer particles are compacted. (b) Initial stage of ultrasonic vibration: by applying ultrasonic vibration, the polymer particles will produce friction in the contact interface under the action of ultrasonic vibration, and the local temperature will rise. (c) Plasticization and cavity filling: viscoelastic heat generation occurs between polymer particles, and the particles gradually melt and fill the cavity. (d) Pressure holding and cooling: the ultrasonic horn exerts a holding pressure in the plasticizing chamber and waits for the polymer melt to cool down. The ultrasonic horn returns to its original position to complete the UPIM process.

This study focused on the friction heating stage of the UPIM process, as shown in Figure 2b. At this stage, the polymer particles slipped due to ultrasonic vibration, and the work done by friction was converted to heat to form local heating of the friction interface. Friction heat generation is the amount of work done by shear stress along the slip direction under the action of ultrasonic vibration.

**Figure 3:**
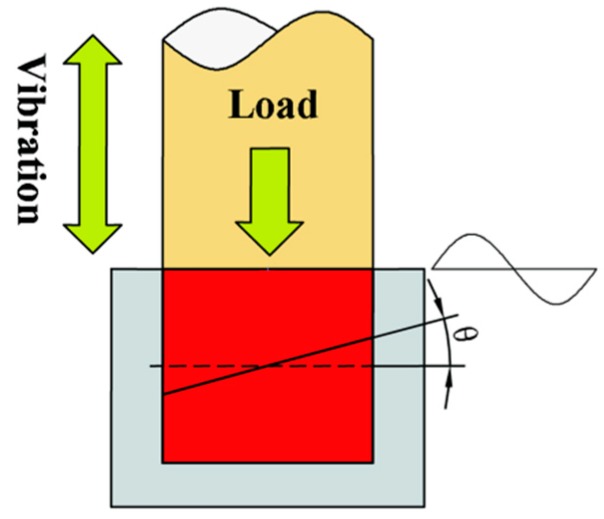
Physical model.

In the UPIM process, frictional heating and the resulting contact temperature can have an essential influence on the plasticizing efficiency. The friction heat generation process between polymer particles is transient (the time required with rising to melt temperature is less than 0.1 s [15]), and it is difficult to measure the temperature change at the contact interface. A polymeric rod model with a constant contact angle *θ* was used to study the friction heating process of UPIM. The physical model is shown in Figure 3. 

The following assumptions were made in the model:

1. Because polymeric particles are cylindrical and spherical, when particles are loaded into the plasticizing chamber, there are three types of contact between particles: point contact, line contact, and surface contact. It is assumed that the contact between particles can be equivalent to line contact, that is to say, when in point contact, the contact point is also line contact under microscopic observation, and when surface contact, it can be considered as an infinite line contact.
2. The transverse propagation of ultrasonic vibration is negligible, and the ultrasonic wave is perpendicular to the polymer without considering the ultrasonic reflection and attenuation.
3. It is assumed that the elastic modulus and friction coefficient of polymeric particles remains unchanged during the friction heating process, and is not affected by temperature and sliding velocity.
4. There is no morphological change in the polymer particles, only considering that the interfacial temperature is lower than the atomization temperature.
5. The heat convection and radiation between horn and polymer are not considered.

### 2.2. Finite Element (FEM) Simulation of Interfacial Friction Heating

The frictional heat generation process of UPIM was studied by using an amorphous polymethyl methacrylate (PMMA, TF8, Mitsubishi Chemical Holdings Group, minato-ku, Tokyo, Japan) rod with a diameter of 10 mm. The friction surface inclination was 30°, the glass transition temperature of the PMMA was 105 °C, and the melt flow temperature was 160 °C. Material parameters are shown in Table 1.

The single factor analysis method was used to analyze the influence of different processing parameters on the temperature rise of friction heating. The ultrasonic frequency ranged from 20 to 40 kHz with a standard value of 20 kHz, and the ultrasonic amplitude ranged from 10 to 30 μm with a standard value of 30 μm.

A temperature-displacement coupled FEM method was used to study the local friction heat generation of the polymer interface during ultrasonic plasticization with a two-dimensional model. Previous studies have shown that the time required for the friction heating temperature of polymer contact interface to reach the melting temperature under ultrasonic vibration is 0.078 s [15]. Therefore the time required for FEM simulation was set to 0.1 s.

The temperature change during friction heating can be estimated by formula (1).

(1) $$T_{total}\;=\;T_{0}\;+\;T_{f}$$

where *T*_0_ is the initial ambient temperature and *T_f_* is the temperature change caused by the friction heat of the contact interface. The governing equation of the transient heat conduction process is shown in formula (2) [22]:

(2) $$pc\frac{\partial T}{\partial t}(x,y,z,t)\;=\;k^{2}▽T(x,y,z,t)\;+\;Q(x,y,z,t)$$

where *Q* is the friction heat generation rate and *Q(x, y, z, t)* = *ημσ(x, y, z, t) v(x, y, z, t)*. Among which *η* is the heat conversion efficiency, *μ* is the friction coefficient, *σ(x, y, z, t)* is the contact stress at coordinates *(x, y, z)* at time *t*, and *v(x, y, z, t)* is the relative slip rate at coordinates *(x, y, z)* at time t. When heat is generated, the boundary conditions of the friction interface between polymers can be formulated as (3):

(3) $$-k\frac{\partial T}{\partial n}|s\;=\;h(T_{s}\;-\;T_{0})$$

where *T_s_* is the temperature of the friction surface and *h* is the heat transfer coefficient.

**Figure 4:**
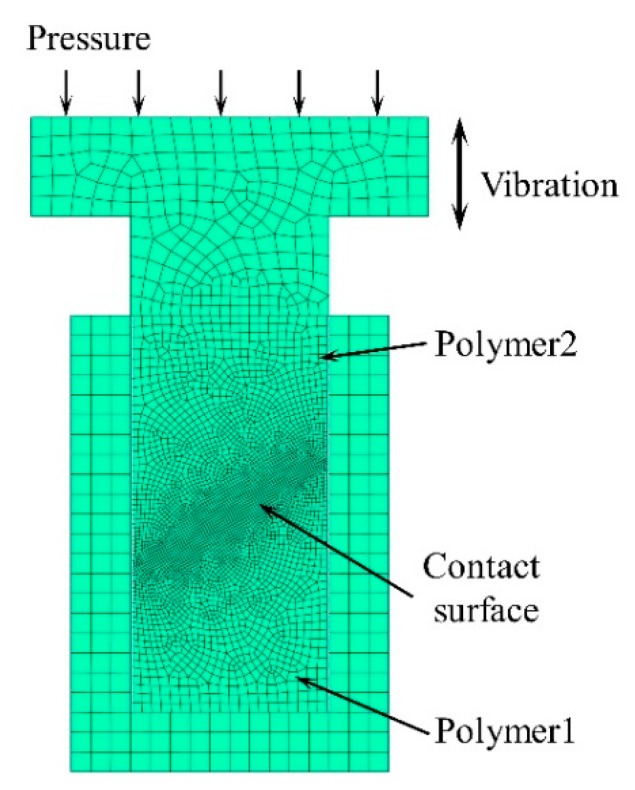
Finite Element model.

The FEM model is shown in Figure 4. To accurately calculate friction heat generation, the mesh of the contact area was refined to a minimum size of 0.2 mm. The boundary conditions in the FEM calculation were as follows:

The ambient temperature was set to 20 °C. The range of ultrasonic frequency of horn was defined as 20–40 kHz and the range of variation was 10 kHz. The range of ultrasonic amplitude was defined as 10–30 μm, and the range of variation was 10 μm.

Contact conditions: the contact surface between polymers was set as friction contact, the friction calculation formula was a penalty, and the friction coefficient was set to 0.4. The other contact surfaces such as polymer and horn, polymer and plasticizing chamber were set as friction contact, without considering heat generation. The whole calculation process was divided into two steps: the first step was the loading and compression process of the ultrasonic horn; the second step was the friction heating process under the action of ultrasonic vibration; in order to accurately simulate the ultrasonic loading period, the minimum time increment was set to 5 μs (1/10 ultrasonic period).

### 2.3. Experimental Research

The changes of frictional surface under ultrasonic vibration are temperature softening, glass transition, high elastic state, and viscous flow state. When the local temperature of the contact interface rises to the melting temperature, the friction heating process is completed. In experimental research, the frictional heating process was studied by using the principle device of Figure 1. The experimental samples were an amorphous PMMA rod with a 30° inclined plane and a diameter of 10 mm. Polymer rods were cut on a saw machine, and the angle was precisely controlled with an angle ruler.

**Figure 5:**
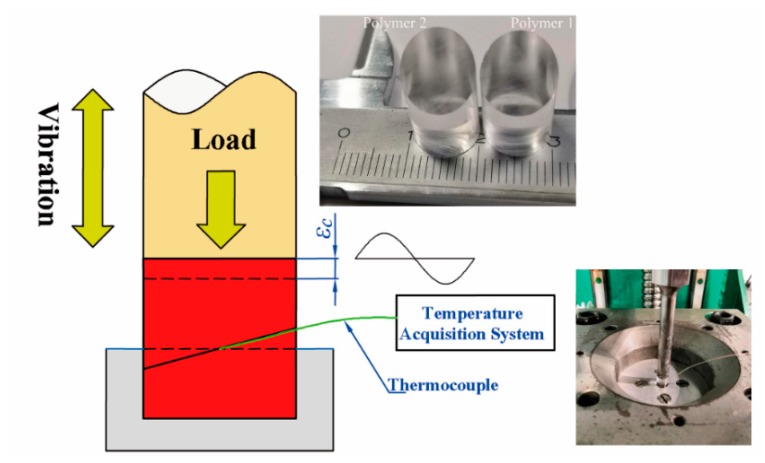
Principle of temperature calculation and measurement.

Temperature measurement: Thermocouple (5TC-TT-K-40-36, Omega, Shanghai, China) was used to measure the temperature change during friction heating. The principle of temperature measurement is shown in Figure 5. A thermocouple probe was arranged on the surface of the polymer contact interface by hot plate welding. Because the ultrasonic horn itself will heat in the process of vibration, considering that the contact between the horn and the polymer will melt part of the material, where the thickness is ***ε****_c_*, the ***ε***_c_ = 1 mm was chosen to compensate for the melting thickness. A data acquisition system developed by ourselves (with Yanhua USB4711A acquisition card, Taiwan, China) was used to collect temperature changes under different process conditions. The end of the thermocouple was not in contact with the friction surface to avoid excessive transient temperature and inaccurate temperature measurement and damaging to the thermocouple.

## 3. Results and Discussion

### 3.1. Finite Element Simulation Results

By importing the data of the material and process parameters and boundary conditions, the temperature curves of the PMMA polymer under different process parameters were calculated. The results of FEM simulation show that the friction heating process of the contact interface was transient during the UPIM process of polymer, and the local interface temperature rise could reach 160 °C within 0.1 s, which was consistent with our previous research [15].

**Figure 6:**
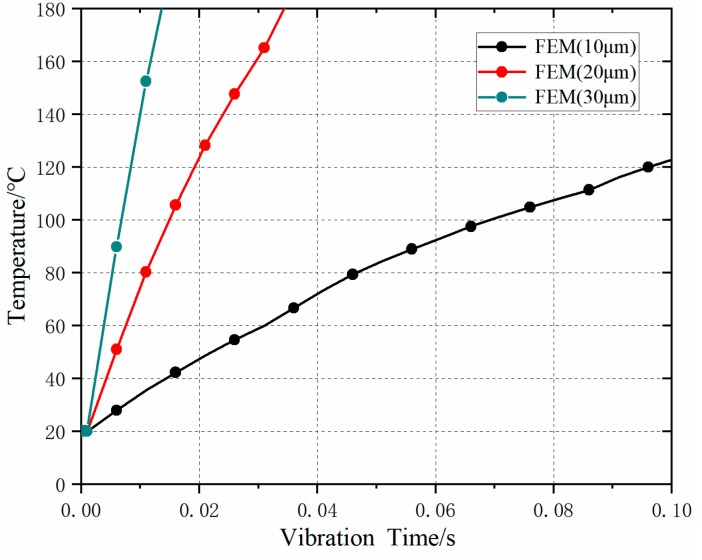
Temperature rise at different ultrasonic amplitudes (*f* = 20 KHz, *F* = 300 N).

The temperature variation curves under different ultrasonic amplitudes are shown in Figure 6. The results show that: with the increase of ultrasonic amplitude, the heat generated by friction increased, and the time to reach the melting state became shorter. When the ultrasonic amplitude was 10 μm, the FEM simulation showed that the melting temperature could hardly be reached in 0.1 s; with the increase of the ultrasonic amplitude, the friction heating temperature changed sharply, and the time to reach the melting temperature became shorter. The increased margin of temperature change rate decreased with the increase in ultrasonic amplitude.

**Figure 7:**
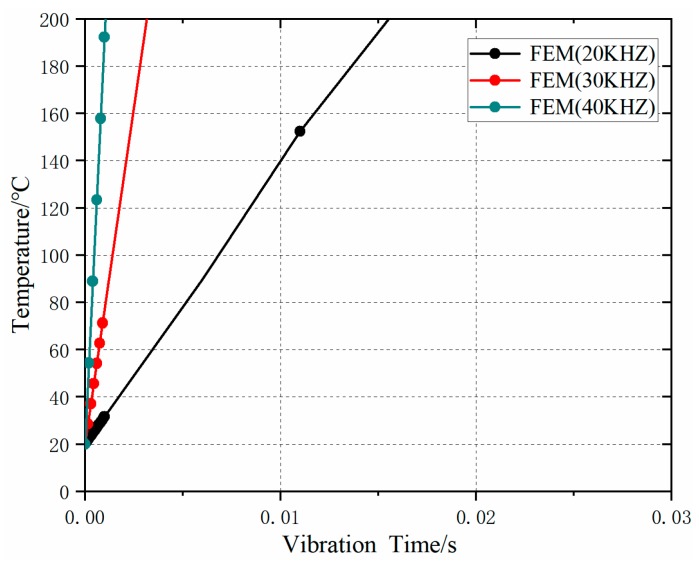
Temperature rise at different ultrasonic frequencies (*u*_0_ = 30 μm, *F* = 300 N).

The temperature variation curves under different ultrasonic frequencies are shown in Figure 7. The results show that: with the increase of ultrasonic frequency, the temperature rising rate increased, the heat generated by friction increased, and the time to reach the melting temperature decreased. When the ultrasonic frequency was greater than or equal to 20 kHz, the melting temperature could be reached within 0.1 s. With the increase of frequency, the increased margin of temperature change rate decreased. From the results of FEM simulation, it can be seen that the influence of ultrasonic amplitude on friction heat generation was more significant than that of ultrasonic frequency (the temperature change was more evident after changing amplitude).

**Figure 8:**
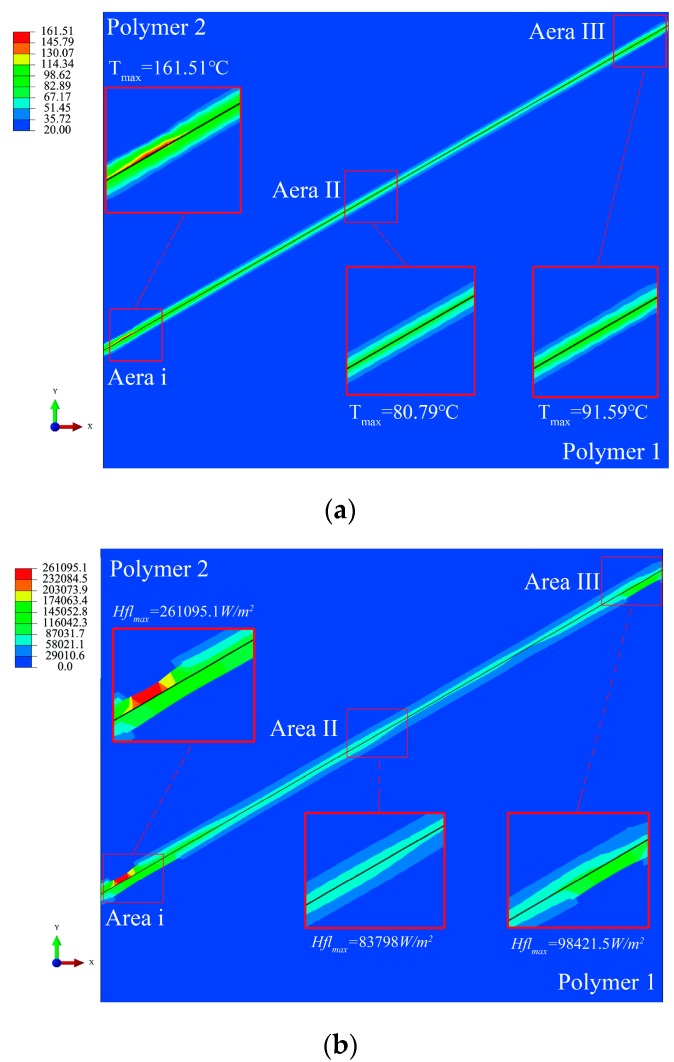
Nephogram of temperature and heat flow distribution at 0.0041 s. (**a**) Distribution of temperature, (**b**) Distribution of heat flux.

The results of FEM simulation show that when the process parameters were *F* = 300 N, *u*_0_ = 30 um and *f* = 20 KHz, the local temperature of the polymer friction block reached 160 °C at 0.0041 s, as shown in Figure 8a. The frictional surface temperature distribution was divided into edge contact zone i, III, and middle contact zone II. The maximum temperature appeared in the edge contact area i, and the temperature of the three calculations was in the order i > III > II. The temperature difference between zone III and II was 10.8 °C. The reasons for the maximum temperature in region i were as follows: polymer block 2 tended to move downward under ultrasonic vibration and the left inner wall of the plasticizing chamber and polymer block 1 blocked its movement trend, resulting in stress and strain concentration in the plasticizing process and temperature rising sharply. Figure 8b is a heat flux distribution nephogram. The results show that the maximum heat flux was 261,095.1 W/m^2^ in region i. The results of heat flux and temperature nephogram show that the temperature change trend in the two edge contact zones was more significant than that in the middle contact zone during ultrasonic plasticization of polymers.

**Figure 9:**
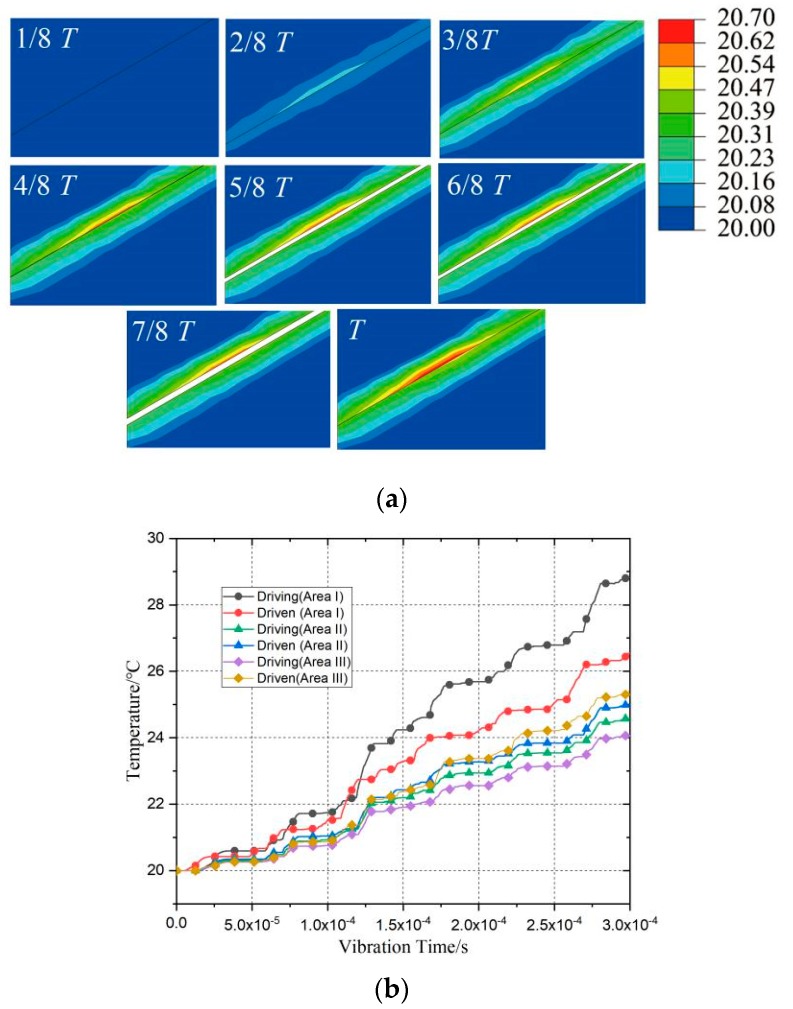
Temperature variation in friction zone of the first period and maximum temperature change of the first 6 periods. (**a**) Finite element analysis results for the temperature change: the temperature distribution during the loading period (T/8~T, 6.25 μs time increment), (**b**) temperature change during the first 6 periods (0~6 T). (area i).

Figure 9a shows the results of temperature distribution in friction region i during the first loading cycle. The results show that the first half cycle (0~T/2) was the loading stage, when the polymer block contacted under vibration displacement, shear deformation occurred on the surface, resulting in a friction heat and temperature rise. In the second half of the cycle (T/2~T), the movement of the horn occurred in the opposite direction. The horn kept in contact with the upper surface of polymer block 2, and polymer block 2 and polymer block 1 were separated. The temperature change of the driven friction surface was more uniform than that of the driving friction surface, and the reason was that the stress concentration occurred in the frictional process in the contact zone at the edge of polymer block 2.

Figure 9b shows the transient temperature trend curve of the nodes with the highest temperature of the friction interface in the three regions of the first six cycles (0–6 T). From the temperature trend diagram, it can be seen that in the first half of each cycle, the temperature tended to increase, and in the second half of the cycle, polymer blocks 1 and 2 were separated without friction, and the temperature rise stagnated. Temperature increases occurred only in the loading stage (nT~ (2n + 1)T/2, n = 0, 1, 2, …). In the unloading stage ((2n + 1)T/2~(n + 1)T, n = 0, 1, 2, …), the temperature of the friction surface will be transferred with the form of heat conduction in the polymer friction interface. Because the heat conduction time was short and the thermal conductivity of the material was low, the temperature of the friction surface remained nearly unchanged during the unloading stage. The temperature variation trend of the driving friction surface and driven friction surface was almost the same. The temperature difference between the master and slave surfaces in region II was small, which indicated that the friction was more uniform. In the first six cycles, the temperature of the driving surface was higher than that of the driven surface. The reason was that the polymer kept interference contact in the plasticizing chamber after being compressed. Under the action of ultrasonic vibration, the frictional zone of edge appears to have stress concentration and friction, which is not uniform under the action of high frequency vibration. Therefore, in the plasticizing chamber, the friction contact line was longer and the friction heat generation of the middle friction zone was more uniform.

**Figure 10:**
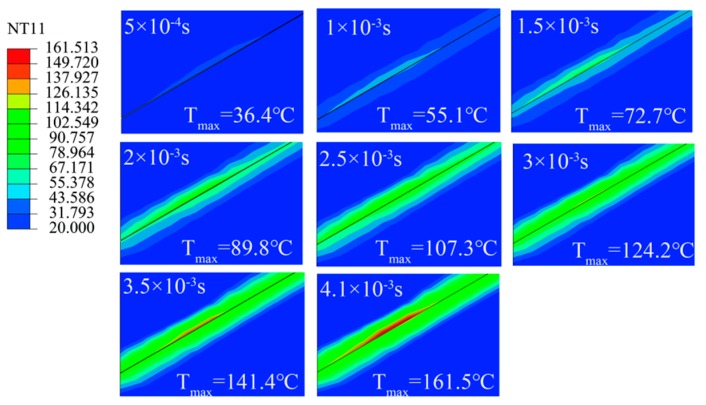
Maximum temperature change in contact zone i.

The temperature change before reaching the melting state in region i is shown in Figure 10. The glass transition temperature was *T_g_* = 105 °C at 0.0025 s and 160 °C at 0.0041 s. Under the same time interval, the temperature change process was linear, and the average change rate was 34,785.7 °C/s. The temperature change of the driven surface was more uniform. 

### 3.2. Experimental Results

**Figure 11:**
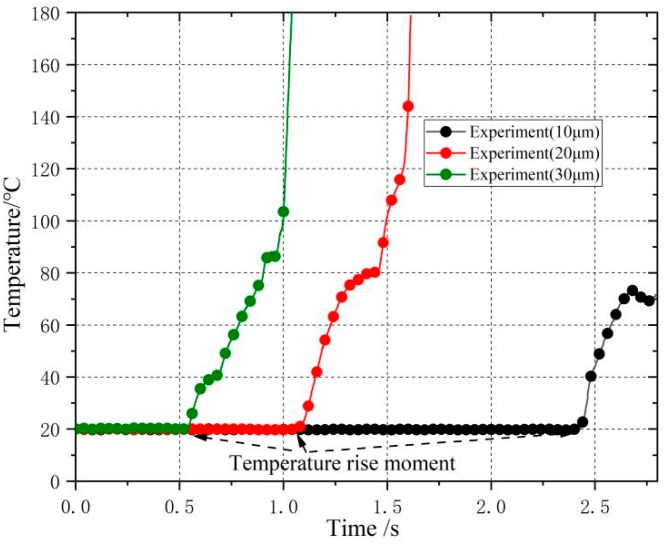
Experiment results of temperature rise curves under different amplitudes.

The comparison of temperature curves under different ultrasonic amplitudes of experimental results is shown in Figure 11. According to the comparison results, the time points of the friction heating temperature rise were different under different amplitudes (for different compaction states). The time points of friction heating under 10, 20 and 30 um were 2.416, 1.064, and 0.541 s, respectively. The larger the ultrasonic amplitude was, the smaller the time point of transient temperature rise of friction heating. Experimental results show the consistent rising trend of temperature with FEM simulation. Both FEM and experimental results show that the friction heating temperature could hardly reach the melting temperature when the ultrasonic amplitude was 10 um. The changing trend of the friction heat generation temperature was the same between FEM and experimental results, and both of them rose sharply in a short time (within 1 s). The experiment results showed that the friction heat temperature changed to the melting temperature time within 0.5 s when the amplitude was 30 um. When the amplitude increased, the time of temperature rise became shorter. The results of temperature measurement by experiment had not risen as sharply as the FEM calculation. The reason may be that the FEM calculation does not consider the change of friction coefficient with the increase of temperature; does not consider the heat exchange with the environment; does not consider the influence of material surface state on the friction heat generation process.

**Figure 12:**
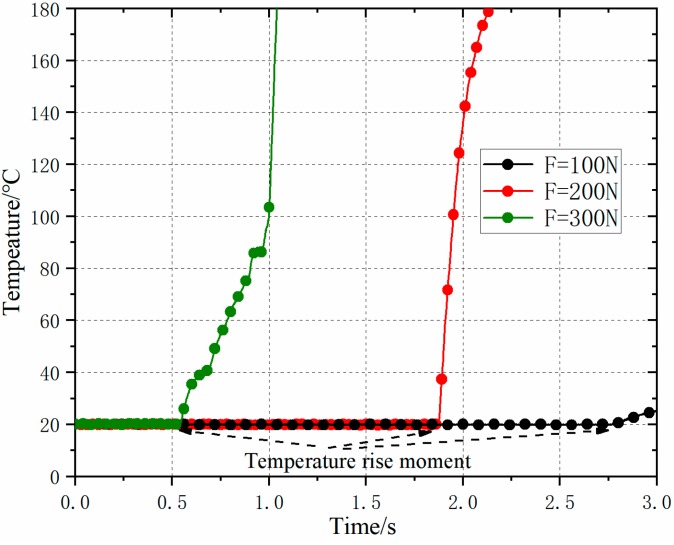
Experiment results of temperature rise curves under different loads.

Under the experimental study, by changing the load of the horn (*f* = 20 KHz, *u*_0_ = 30 um), the temperature change curve is shown as Figure 12. The results show that the time points of transient temperature rise were different under different horn loads. The larger the horn load was, the smaller the time point of transient temperature rise. When the load was 100 N, the friction heating temperature lasted longer, and the plasticizing efficiency was low. Therefore, to ensure plasticizing efficiency, the horn load should be higher than 100 N.

## 4. Conclusions

In this study, the friction heat generation of UPIM process was studied by FEM simulation and experiment. The friction heat generation process of polymer interface under ultrasonic vibration was analyzed, and the temperature rise was predicted. Based on the FEM simulation results, the effects of different UPIM process parameters on the temperature change of interfacial friction heat generation of polymer were studied. According to the results, it could be concluded that the friction heat generation process of the UPIM process was transient (the friction heat generation of the local area to melting temperature can be completed in 0.1 s), and the temperature change in the edge contact zone was more drastic than that in the middle contact zone. The proposed FEM simulation process is helpful for explaining the friction heat generation mechanism in the UPIM process, to control the UPIM process and improve the quality of UPIM plastic parts.

The temperature change of friction heat generation in the UPIM process was also affected by many factors such as the roughness of the contact surface, the compaction state of the friction surface and the moisture content of the material. The research on friction heat generation under different states of the material can be carried out in the later stage. A more accurate estimation of friction heat generation can be obtained by establishing the mathematical model in a future study.

## Figures and Tables

**Table 1:** Parameters of the polymethyl methacrylate.

Density [Kg/m^3^]	Thermal Conductivity [W/m °C]	Specific Heat Capacity [J/Kg °C]	Glass Transition Temperature [°C]	Elastic Modulus [GPa]	Poisson’s Ratio
1.166	0.18	1828	105	3.3	0.345
